# Fungal Fermented Palm Kernel Expeller as Feed for Black Soldier Fly Larvae in Producing Protein and Biodiesel

**DOI:** 10.3390/jof8040332

**Published:** 2022-03-23

**Authors:** Chin Seng Liew, Chung Yiin Wong, Eman A. Abdelfattah, Ratchaprapa Raksasat, Hemamalini Rawindran, Jun Wei Lim, Worapon Kiatkittipong, Kunlanan Kiatkittipong, Mardawani Mohamad, Peter Nai Yuh Yek, Herma Dina Setiabudi, Chin Kui Cheng, Su Shiung Lam

**Affiliations:** 1 Department of Fundamental and Applied Sciences, HICoE-Centre for Biofuel and Biochemical Research, Institute of Self-Sustainable Building, Universiti Teknologi PETRONAS, Seri Iskandar 32610, Perak Darul Ridzuan, Malaysia; chinseng93@gmail.com (C.S.L.); chung_16001113@utp.edu.my (C.Y.W.); ratchaprapa_20000290@utp.edu.my (R.R.); hemamalini_20001043@utp.edu.my (H.R.); 2 Entomology Department, Faculty of Science, Cairo University, Cairo 12613, Egypt; ealaaeldein@sci.cu.edu.eg; 3 Department of Chemical Engineering, Faculty of Engineering and Industrial Technology, Silpakorn University, Nakhon Pathom 73000, Thailand; 4 Department of Chemical Engineering, School of Engineering, King Mongkut’s Institute of Technology Ladkrabang, Bangkok 10520, Thailand; kunlanan.kia@kmitl.ac.th; 5 Faculty of Bioengineering and Technology, Universiti Malaysia Kelantan, Jeli Campus, Jeli 17600, Kelantan, Malaysia; mardawani.m@umk.edu.my; 6 Centre for Research of Innovation and Sustainable Development, Department of Engineering and Technology, University College of Technology Sarawak, Sibu 96000, Sarawak, Malaysia; peter.yek@ucts.edu.my; 7 Henan Province Engineering Research Center for Biomass Value-Added Products, School of Forestry, Henan Agricultural University, Zhengzhou 450002, China; lam@umt.edu.my; 8 Faculty of Chemical and Process Engineering Technology, College of Engineering Technology, Universiti Malaysia Pahang, Lebuhraya Tun Razak, Gambang, Kuantan 26300, Pahang, Malaysia; herma@ump.edu.my; 9 Center for Catalysis and Separation (CeCaS), Department of Chemical Engineering, College of Engineering, Khalifa University of Science and Technology, Abu Dhabi P.O. Box 127788, United Arab Emirates; cheng.kui@ku.ac.ae; 10 Pyrolysis Technology Research Group, Higher Institution Centre of Excellence (HICoE), Institute of Tropical Aquaculture and Fisheries (AKUATROP), Universiti Malaysia Terengganu, Kuala Nerus 21030, Terengganu, Malaysia

**Keywords:** black soldier fly larvae, palm kernel expeller, *Rhizopus oligosporus*, fermentation, protein, biodiesel

## Abstract

Being the second-largest country in the production of palm oil, Malaysia has a massive amount of palm kernel expeller (PKE) leftover. For that purpose, black soldier fly larvae (BSFL) are thus employed in this study to valorize the PKE waste. More specifically, this work elucidated the effects of the pre-fermentation of PKE via different amounts of *Rhizopus oligosporus* to enhance PKE palatability for the feeding of BSFL. The results showed that fermentation successfully enriched the raw PKE and thus contributed to the better growth of BSFL. BSFL grew to be 34% heavier at the optimum inoculum volume of 0.5 mL/10 g dry weight of PKE as compared to the control. Meanwhile, excessive fungal inoculum induced competition between BSFL and *R. oligosporus*, resulting in a reduction in BSFL weight. Under optimum feeding conditions, BSFL also registered the highest lipid yield (24.7%) and protein yield (44.5%). The biodiesel derived from BSFL lipid had also shown good compliance with the European biodiesel standard EN 14214. The high saturated fatty acid methyl esters (FAMEs) content (C12:0, C14:0, C16:0) in derived biodiesel made it highly oxidatively stable. Lastly, the superior degradation rate of PKE executed by BSFL further underpinned the sustainable conversion process in attaining valuable larval bioproducts.

## 1. Introduction

In recent years, the reported studies associated with the biodiesel derived from black soldier fly larvae (BSFL) have intensified, offering a new and sustainable feedstock to the renewable energy industries. In this regard, various organics, usually in the form of wastes such as sewage sludges, agricultural by-products, and food wastes, have been administered to BSFL in challenging the capability of larvae to valorize and later assimilate wastes into its body biomass [1,2]. Furthermore, the exploitation of organic wastes as larval feed could mitigate the untoward environmental impacts should the wastes be disposed of indiscriminately. In such a situation, BSFL serve as an agent to reduce organic wastes, managing the waste volume in an environmentally friendly manner, whilst producing a useful larval biomass feedstock for biofuel industries. Shortcomings arise when BSFL confront its valorization capability limits, i.e., when the larvae are fed with recalcitrant organics that contain a high composition of lignocellulosic materials or organic wastes impoverished of essential nutrients. In both cases, the growth of BSFL will be retarded, and there is a high possibility the larvae will require a longer duration to reach its higher instars, should the larvae survive until the pupation stage. In enriching the organic wastes of this class, the fermentation process has been introduced to fortify the nutritional contents of the larval feed. Indeed, various fermented larval feeds have been explored to grow BSFL positively, and these include maize straw, coconut waste, pulp and paper bio-sludge, dried distiller grains, rice straw, duck manure, etc. [3,4,5,6,7].

In general, fermentation is a process carried out under aerobic or anaerobic conditions by a single or group of microorganisms that catalyze the breakdown of biopolymers in the organics, while producing secondary metabolites as well as fulfilling other physiological activities of the fermenters [8]. The fermentation process has been widely employed, mostly in food industries to produce fermented foods, including bread, kimchi, soy sauce, tempeh, cheese, etc. Accordingly, different inoculations of microorganisms, e.g., bacteria and fungi, are introduced into the mediums during fermentation [9]. The prime intention of fermentation is to help unleash the nutrients that are naturally found in the starting materials to become more accessible and digestible for the later consumers upon the completion of the fermentation process [10]. On the other hand, besides food industries, the fermentation process has also been applied in bioprocesses such as the bioremediation and biodegradation of hazardous organic materials, the bioconversion of biomasses, the biotransformation of agricultural residues, and the biopulping and synthesis of valuable metabolites, e.g., antibodies and enzymes [11].

*Rhizopus oligosporus* is a zygomycete that has been broadly used to ferment soybean, turning it into tempeh [12]. Through fermentation, the protein-nitrogen content will decrease and amino-nitrogen and ammoniacal nitrogen concentrations will conversely increase in soybean product after having been altered by the proteolytic activities of *R. oligosporus* [13]. In fact, *R. oligosporus* is capable of exuding enzymes that can hydrolyze the protein, lipid, and starch, as well as change the physical and functional properties of the fermented soybean [14]. The presence of *R. oligosporus* also inhibits the growth of pathogenic bacteria such as *Helicobacter pylori* that could otherwise cause stomach inflammation, chronic gastritis, and even worse, gastric cancer. [15]. Moreover, researchers found that the compositions of protein and protein solubility, in vitro protein digestibility, and essential amino acids in tempeh increase after the fermentation process [16], signifying nutritional enrichment. The solid-stage fermentation by *R. oligosporus* was also found to fortify the nutritional indicators, including the protein efficiency ratio, protein digestibility, and corrected amino acid score in chicken pea-fermented flour [17]. In this regard, ideally, *R. oligosporus* is a good fermenter, and therefore was chosen as an inoculant in this study.

Palm kernel expeller (PKE) is a by-product generated by palm oil mills and is currently serving as the feed for ruminants and broilers in Malaysia [18]. It has been reported that it was possible to feed absolute PKE to the ruminants without any negative impacts on growth, while also providing ample amounts of calcium and vitamins [19]. On the other hand, the incorporation of PKE into broilers’ feed has shown prebiotic features and lowered the *Escherichia coli* population in the digesta [20]. PKE is well-known as an acceptable feed replacement for ruminants’ and non-ruminants’ diets since it serves as a good protein source. Furthermore, it is cost effective and abundantly available in Malaysia throughout the year [18,19]. Thus, the main objective of this study was to enhance the nutritional characteristics of PKE not only to suit farm animals, but also the palatability of BSFL, regarding the excessive quantities produced from palm oil mills, since Malaysia is the second-largest producer of palm oil in the world [21]. This was achieved through prior fermentation of PKE with different inoculations of *R. oligosporus* spore suspensions before BSFL feeding. Accordingly, BSFL performances, including the valorization of fermented PKE as well as larval biochemical productions when feeding with different fermented PKE, were unveiled in this study. The enriched PKE feed was expected to promote the growth of BSFL, and subsequently, translate into high BSFL-based biodiesel production.

## 2. Materials and Methods

### 2.1. Preparation of Rhizopus oligosporus Spore Suspension

To produce the *R. oligosporus* spore suspension, Potato Dextrose Broth (Sigma-Aldrich) and Potato Dextrose Agar (Merck) were prepared beforehand. The sterile Potato Dextrose Broth was prepared by adding 6 g of the dehydrated medium into 250 mL of distilled water and autoclaved at 121 °C for 15 min. The sterile Potato Dextrose Agar was prepared by adding 39 g of the dehydrated medium into 1 L of distilled water followed by autoclaving at 121 °C for 15 min. *R. oligosporus* activation was carried out by inoculating 20 g of the dried culture of *R. oligosporus* (Raprima Brand) into the 250 mL sterile Potato Dextrose Broth and leaving it for incubation in an incubator shaker operating at 180 rpm and 30 °C for 48 h. Then, 1 mL of the activated *R. oligosporus* culture was transferred into the sterile Potato Dextrose Agar, spread, and incubated at 30 °C for approximately 7 days until the presence of black spores could be observed. For *R. oligopsorus* spore harvesting, a desired amount of sterile distilled water was slowly added to the agar plate, and the spores were dislodged using a sterile inoculating loop. The final concentration of the spore suspension was adjusted to approximately 1.0 × 10^6^ spores per mL as determined from the cell counting plate [22,23]. All these steps were carried out under aseptic conditions to prevent contamination.

### 2.2. Rearing of Black Soldier Fly Larvae

Freshly laid BSF eggs were procured from MLF Ingredient Sdn Bhd located in Johor, Malaysia. The eggs were immediately transported and transferred to a sterile Petri dish with the moisture controlled by wet filter paper. The dish was left for incubation at 27 °C until the neonate had closed after about 4 days. The newly hatched larvae were reared with raw palm kernel expeller waste up to 6 days old prior to their use in the experiments.

The initial moisture of PKE was adjusted to 70% by adding adequate sterile distilled water. The fermentation of PKE was carried out by adding different volumes (0.1, 0.5, 1, 2, 3, 4, 5 mL) of the *R. oligosporus* spore suspension into each 10 g moisture-adjusted PKE sample. The samples were then homogenized by shaking and left for incubation at 30 °C for 72 h until the presence of white mycelium could be observed.

Upon completion of fermentation, 20 6 -day-old BSFL were allowed to inoculate in 10 g of fermented PKE containing various *R. oligosporus* inoculum volumes. A 10 g control of PKE that was free from *R. oligosporus* spores was also set up and inoculated with 20 6-day-old BSFL. Throughout the larval rearing period, PKE moisture was maintained at 60–70% for all setups. The rearing of BSFL was terminated when half of the larvae had reached the late 5th instar stage as identified from its body colors and head sizes [24]. The separation of BSFL from the PKE medium was performed, and the harvested BSFLs were washed with distilled water, deactivated at −20 °C, and then dried at 60 °C to a constant weight. The residues of PKE were also separately dried at 105 °C until reaching constant weights.

### 2.3. Growth Performances of Black Soldier Fly Larvae

The growth of BSFL was measured in terms of biomass gained after the rearing period. The efficiency of the conversion of digested feed (ECD) was also recorded to signify the efficacy of ingested PKE being assimilated into larval biomass [4]. Finally, the treatment of PKE in terms of a reduced quantity was measured by the degradation rate.

Biomass gained (g) = Final BSFL dry weight (g) − Initial BSFL dry weight (g)(1)

ECD (%) = Biomass gained (g)/Total feed consumed (g) × 100% (2)

Degradation rate (%) = (Initial PKE dry weight (g) − Final PKE dry weight (g))/(Initial PKE dry weight (g)) × 100%(3)

### 2.4. Biochemical Analyses

#### 2.4.1. Lipid

Using petroleum ether as a solvent, the lipid from BSFL biomass was extracted via the immersing technique. Initially, 100 mg of ground larval biomass was added with 20 mL of petroleum ether and agitated for 24 h. To separate the larval lipid from petroleum ether, the mixture was filtered through filter paper, and the filtrate was dried in a rotary evaporator. Finally, the weight of the extracted lipids was recorded and used to compute the BSFL lipid yield and lipid productivity.

Lipid yield (%) = (Dry weight of extracted lipid (g))/(Dry weight of BSFL biomass (g)) × 100%(4)

#### 2.4.2. Protein

The protein content of BSFL biomass was calculated by multiplying the nitrogen content of the larvae by a factor of 6.25 [25]. Using the Perkin Elmer CHNS/O Elemental Analyzer 2400 Series II, the larval nitrogen content and PKE nitrogen content were determined via the Dumas combustion technique. Initially, 1 mg of ground BSFL biomass or PKE was encapsulated in tin foil. The sample was then oxidized at 965 °C in the combustion chamber before being reduced to 640 °C in the reduction chamber. The BSFL protein yield was subsequently calculated as below.

Protein yield (%) = Nitrogen content in BSFL biomass (%) × 6.25 (5)

#### 2.4.3. Fatty Acid Methyl Ester

A two-step reaction with methanol yielded a variety of fatty acid methyl esters (FAMEs) from the extracted BSFL lipid, specifically acid-catalyzed esterification, followed by base-catalyzed transesterification. The procedures were carried out according to Wong et al. [4]. The Shimadzu GC-2010 plus equipped with a flame ionization detector and a polythene glycol capillary column BPX-BD20 (30 m × 0.32 mm × 0.25 m) was used to analyze the larval FAMEs mixture. Finally, the larval FAME profile was calculated as reported by Lim et al. [26].

FAME in biodiesel (%) = (A_FAME_/A_ISTD_) × (C_ISTD_ × V_ISTD_)/m × 100% (6)

where A_FAME_ represents the peak area of a specific FAME species, A_ISTD_ represents the peak area of the internal standard, C_ISTD_ represents the concentration of the internal standard (1.00 mg/mL), V_ISTD_ represents the volume of the internal standard (1 mL), and m represents the dry weight of biodiesel mixed with the internal standard (mg).

## 3. Results and Discussion

### 3.1. Growth of BSFL Fed with Various Fermented PKE

**Figure 1:**
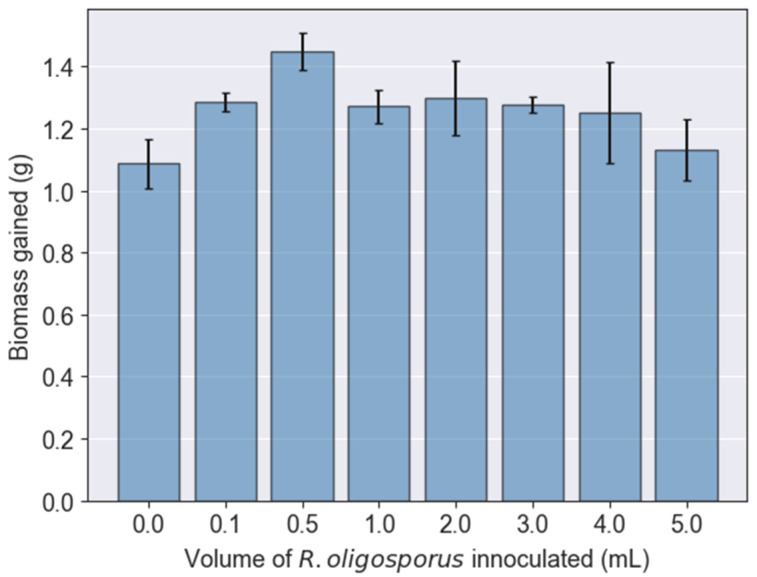
Total weight of biomass gained by black soldier fly larvae fed palm kernel expeller containing various inoculation volumes of *R. oligosporus*.

The experimental results indicated that PKE could be used as a substrate for feeding BSFL. For the control set in which no *R. oligosporus* was inoculated, the weight gained by BSFL was 1.08 g for a total of 20 mature larvae. In addition, as shown in Figure 1, the inoculations with *R. oligosporus* could clearly improve the growth of BSFL (*p* < 0.05), across all tested volumes. The optimum volume as illustrated from the figure was 0.5 mL. When the *R. oligosporus* population was increased gradually from 0 to 0.5 mL, BSFL could attain a maximum weight gain of 1.45 g. Exceeding the optimum volume, the weight gained by BSFL abruptly dropped by about 12% to 1.27 g. The subsequent addition of a higher fungal volume for fermentation did not significantly affect the growth of BSFL, with their weight gain hovering around 1.25 to 1.30 g. However, when excessive *R. oligosporus* was added at 5 mL, the weight gained by BSFL again dropped drastically to 1.13 g, which was close to the control set.

**Figure 2:**
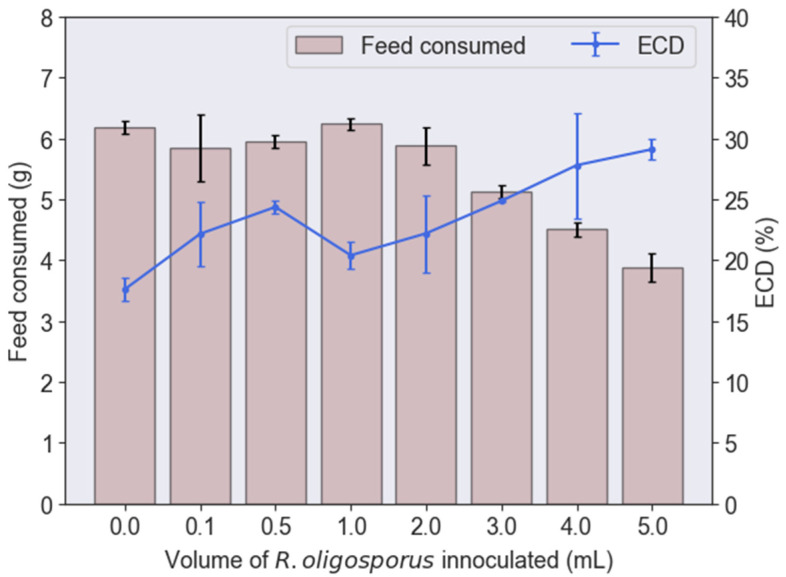
Total feed consumed and the efficiency of conversion of digested feed (ECD) by black soldier fly larvae fed with palm kernel expeller containing various inoculation volumes of *R. oligosporus*.

When the volume of fungal inoculation was increased from 0 to 0.5 mL, the ECD also increased from 17.61% to 24.37% (Figure 2). However, it was notable that the feed consumed by BSFL remained almost constant at this stage. In other words, BSFL were consuming a similar amount of feed but managed to grow heavier when the volume of fungal inoculation increased from 0 to 0.5 mL. This indicated that the fermentation process was effective in improving the palatability of PKE, in which it enriched the nutritional content available per unit of feed for growing BSFL. Lateef et al. [27] had proven that fermentation using *R. stolonifer* could also reduce the crude fiber in PKE by 44.5%, while increasing the crude protein content by 33.2%. This also incontrovertibly justified the better growth of BSFL while being administered fermented PKE with increasing fungal volume from 0 to 0.5 mL (Figure 1).

Beyond the optimum volume of 0.5 mL, the ECD dipped to 20.40%. The dip occurred at 1 mL of fungi with the same amount of feed consumed, but a lower BSFL biomass gained as opposed to 0.5 mL. This could signify that the *R. oligosporus* added was overwhelming. The excessive population of *R. oligosporus* would compete for nutrients with the BSFL. Furthermore, this could accelerate the exhaustion of nutritional contents within the feed, resulting in an impoverished substrate that was no longer enriching for BSFL consumption. This was also exemplified by early work [27] in which the lipid content in fermented PKE was reduced by 15.4% after the fermentation process carried out by *R. stolonifer*. Another plausible rationale could be the imbalance of nutrients in the PKE substrate. Raw PKE consisted of 16% protein, 9.3% fat, and the remaining 73% was neutral detergent fiber (namely, lignin, cellulose, and hemicellulose) [28]. However, with the aid of excessive *R. oligosporus*, a similar phenomenon concerning the enhancement of crude protein content could occur [27], which would further elevate the total composition of crude protein in PKE. In contrast, besides having a balanced amount of protein, the recommended BSFL substrate should consist of plenty of non-fiber carbohydrates as they can be easily digested and converted into larval body lipids [29].

**Figure 3:**
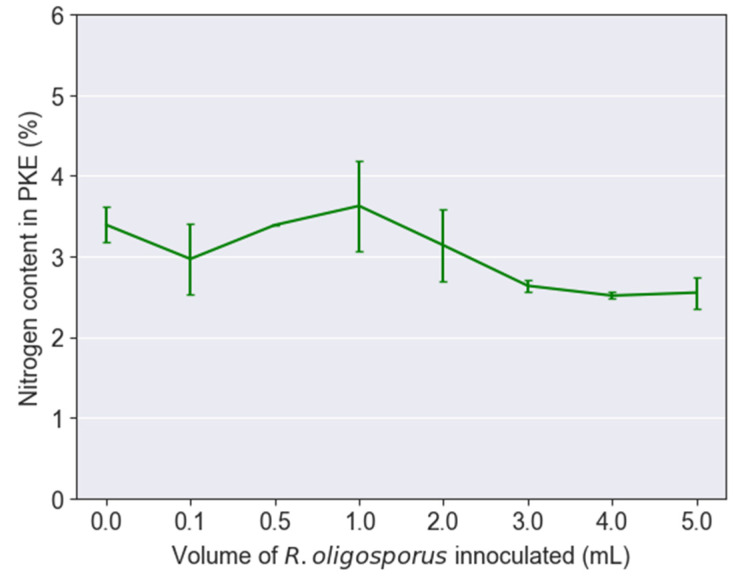
Nitrogen contents in palm kernel expeller residues after feeding to black soldier fly larvae.

A substrate that was only high in protein and low in carbohydrates had been proven to be underperforming in growing BSFL effectively. Lim et al. [26], for instance, had shown that beyond the optimum protein content required, BSFL growth would present a reverse trend as the larvae performed the proteinogenic nitrogen detoxification process in order to survive the high protein content in the feed. This detoxification process was energy-intensive, and hence, would stunt or even reverse the growth rate of BSFL. The high intake of protein by BSFL with 1 to 5 mL inoculum volumes can be seen from Figure 3, where the nitrogen contents of PKE residue dropped by nearly 28%, i.e., from 3.63 to 2.55 wt%. The reduction in nitrogen contents from the PKE residue was directly translated into the intake of nitrogen in the form of protein by BSFL.

Interestingly, the ECD climbed from a low of 20.40% to 29.11% when the volume of *R. oligosporus* inoculated was increased from 1mL to 5mL. This occurred primarily because of the reduction in feed consumed, while the weight of biomass gained remained almost constant. When the volume of *R. oligosporus* inoculated was increased from 1mL to 5mL, the feed consumed by BSFL dropped gradually from around 6.24 g to 3.88 g (*p* < 0.05). Similar to other microorganisms, *R. oligosporus* perform respiration to generate energy to sustain their cellular activities. At the same time, respiration also produces carbon dioxide and water as by-products. Hence, the presence of an excessively high *R. oligosporus* volume in PKE feed could result in intense aerobic respiration, which resulted in increasing the water content in the substrate. Hence, when volumes of *R. oligosporus* higher than 1 mL were inoculated, BSFL could have been ingesting the same amount of substrate as before, but with higher water content. This could have been the cause of the declining amount of feed consumed when *R. oligosporus* was increased from 1 to 5 mL, since the feed consumed was measured in terms of dry weight.

### 3.2. Protein and Lipid to Biodiesel from BSFL Fed with Various Fermented PKE

**Figure 4:**
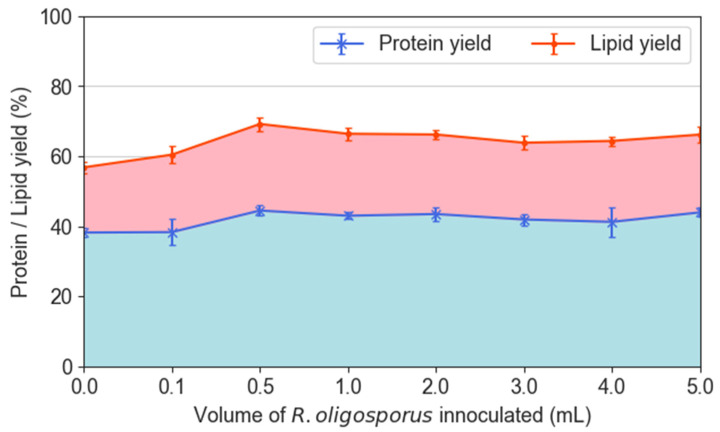
Protein and lipid yields from black soldier fly larvae fed palm kernel expeller containing various inoculation volumes of *R. oligosporus*.

The ANOVA analysis proved that the presence of *R. oligosporus* enhanced the protein yield (*p* < 0.05) and lipid yield (*p* < 0.05) as compared with non-fermented PKE. As illustrated in Figure 4, both protein and lipid yields presented a similar trend when the volume of *R. oligosporus* was increased from 0 to 0.5 mL. Protein yield increased from 38.2% to 44.5%, which was the highest protein yield recorded across the entire set of samples. Subsequently, the protein yield of BSFL registered a slight decline with more fungi introduced but did not drop further to below 41%. On the other hand, the lipid yield increased from 18.6% to a maximum of 24.7% at a 0.5 mL fungal volume. Again, the lipid yield declined slightly with more fungal inoculation, but still registered a higher lipid yield in comparison with the unfermented PKE. The protein and lipid yields in Figure 4 were in conformity with the findings from the previous section in which a 0.5 mL fungal inoculation was singled out as the optimum inoculum size that could achieve the highest larval biomass gained. Accordingly, not only was the maximum BSFL weight gained, but it also had the highest protein and lipid yields as opposed to the other inoculum sizes. At the optimum inoculation volume of 0.5 mL, the protein yield had increased by 16.5%, the lipid yield had increased by 32.8%, and the biomass gained had increased by 34.2%.

In comparison with the previous work on BSFL rearing administered with fermented coconut endosperm waste, the lipid yield of BSFL always ranged around 40%, which was consistently higher than the protein yield [23,30]. However, among all the inoculum sizes in this work, the opposite was observed. The protein yields had been consistently approximately two times higher than the respective lipid yields from BSFL upon feeding with PKE. This could primarily stem from the different substrates being fed to BSFL, proffering a different spectrum of nutrients. The mature coconut endosperm had been reported to consist of 28.7 wt% carbohydrates, 63.2 wt% fat, and only 6.28 wt% protein [31]. This means the coconut endosperm is a highly energy-dense substrate due to the high amounts of fat and carbohydrate. Meanwhile, as stated early, PKE had only 16 wt% protein and 9.3 wt% fat that were easily digestible, while the remaining 73 wt% existed as neutral detergent fiber (namely, lignin, cellulose, and hemicellulose) [28,32]. Although the high protein composition in PKE had boosted the BSFL protein yield, the lack of easily digestible carbohydrates could be one of the reasons for low BSFL lipid yield in this work.

**Figure 5:**
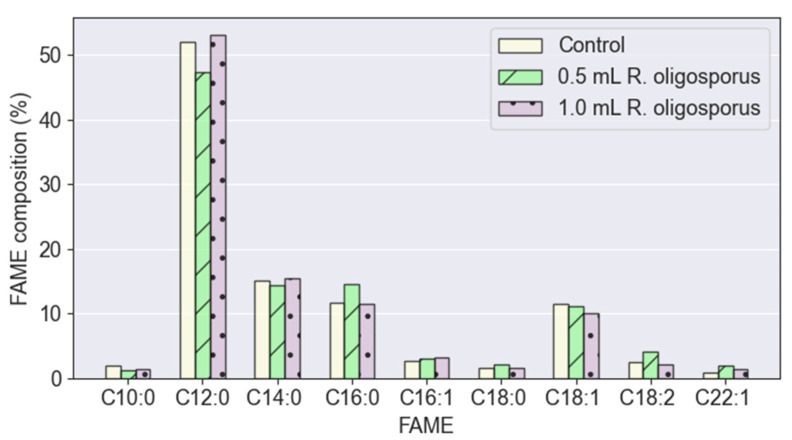
FAME profiles derived from black soldier fly larvae fed with palm kernel expeller containing various inoculation volumes of *R. oligosporus*.

The FAME profiles derived from BSFL fed the controlled PKE (0 mL), the optimum inoculum size PKE (0.5 mL), and the random inoculum size PKE (1.0 mL) were investigated. From Figure 5, it ca be deduced that the fermentation process executed by *R. oligosporus* had no significant effect on the composition of FAME upon feeding BSFL with PKE. The C12:0 was consistently the highest FAME at approximately 47–53 wt% of the total FAME composition regardless of either the absence or presence of *R. oligosporus.* This was then followed by C14:0, C16:0, and C18:1. These four types of FAMEs made up 87–90 wt% of the entire FAME composition. The sum of saturated FAMEs ranged from 79–83 wt%, thereby making BSFL-derived biodiesel highly oxidatively stable. In comparison, other typical biodiesels derived from soybean, rapeseed, and oil palm were inferior in the sense that they had a low saturated FAME composition of 7.4–49 wt% [33]. Their FAME compositions and respective saturated FAME contents are summarized in Table 1. Furthermore, the larval biodiesel attained in this work also met the European specification (EN-14214) where the poly-unsaturated FAME (≥4 π bonds) was lower than 1 wt% [33]. The linolenic acid methyl ester content (2.17–4.02 wt%) was also well below the standard of 12 wt%. Even though the larval biodiesel produced possessed desirable traits, the *R. oligosporus*-fermented PKE seemed to be a more suitable substrate for producing high-protein-yield larvae instead of high-lipid-yield larvae to produce biodiesel.

### 3.3. Degradation Rate of PKE by BSFL

**Figure 6:**
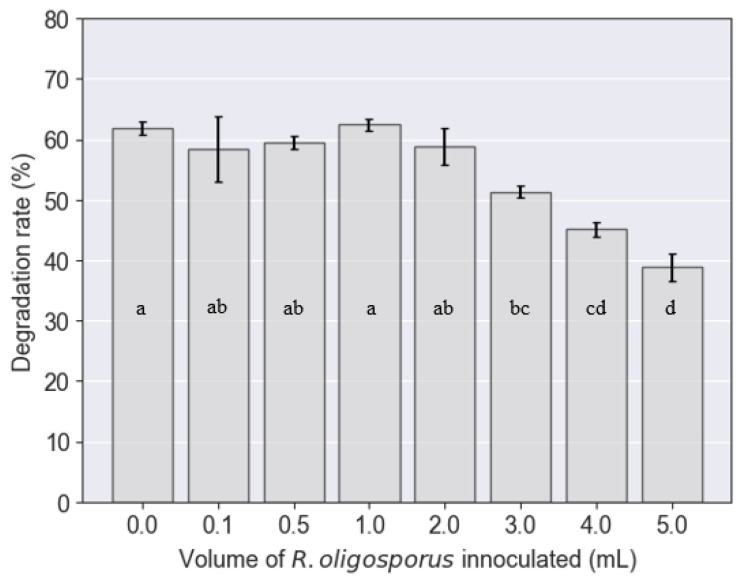
Degradation rates of palm kernel expeller executed by black soldier fly larvae upon inoculation with various volumes of *R. oligosporus*.

The PKE degradation rate was influenced by the inoculation volume of *R. oligosporus* (*p* < 0.05). Nonetheless, the post-Tukey test results as demonstrated in Figure 6 evidenced that there was little difference among the first few samples in which the inoculation volumes were less than 2 mL since they belonged to the same category. Thereafter, the higher inoculation volumes had manifested a significant difference in degradation rates. The degradation rates were found to decline when the fungal inoculation was more than 2 mL. Initially, the degradation rates of PKE fluctuated around 58.4–62.4%. However, when the inoculation volumes increased to 3, 4, and 5 mL, the degradation rates dropped drastically to 51.3%, 45.1%, and, finally, 38.8%, respectively. The decline in degradation rates could be attributed to the wetter substrate conditions at higher inoculum volumes. The presence of denser populations of *R. oligosporus* at higher inoculation volumes could have resulted in higher respiration rates, generating more moisture within the PKE substrate. This resulted in a moister and more diluted substrate, engendering BSFL to feed on less PKE measured as dry matter. In comparison with other substrates that had been fed to BSFL, the degradation rates, ranging from 58.4–62.4%, that were achieved by the control and samples with lower inoculum volumes (<2 mL) were deemed attractive, having been positioned at relatively high values. The degradation rates of other known substrates for BSFL treatments are shown in Table 2 in which the PKE was found comparable to coconut endosperm waste and soybean curd residue, whilst being higher than wheat bran and food waste.

## 4. Conclusions

The fermentation of PKE through the inoculation of *R. oligosporus* evidently enhanced the growth of BSFL. The optimum inoculum volume was found to be 0.5 mL/10 g dry weight of PKE. At this inoculum condition, the biomass gained by BSFL improved remarkably by 34%. The highest lipid and protein yields from BSFL were recorded at 44.5% and 24.7%, respectively, which transpired under the optimum inoculum volume as well. The biodiesel quality derived from BSFL, on the other hand, was not significantly affected by the presence or absence of the fermentation process. Accordingly, biodiesels were all of good quality, oxidatively stable, and fulfilled the FAME requirements as decreed by European biodiesel standard EN 14214. Despite positive enhancements from PKE fermentation for BSFL growth and later larval biodiesel characteristics, the resulting BSFL biomass had an almost two-fold higher protein yield than lipid yield. While more future work can be conducted to enrich the carbohydrate and lipid contents in PKE substrate to boost the lipid yield from BSFL, thus far, fermented PKE seems to be more ideal for the production of high protein content rather than larval lipid for biodiesel. The high protein accumulation via rapid BSFL growth was also underpinned by the high PKE degradation rate via assimilation into the larval body weight, heralding PKE palatability for BSFL feeding as opposed to other substrates. The high content of protein in larval biomass could then be applied as an alternative nutritional food for the substitution of conventional animal feed. Meanwhile, biodiesel-derived larvae could be further improved in terms of lipid yield and utilized as a new generation of biodiesel feedstock in energy industries due to their good quality of FAME compositions.

## Figures and Tables

**Table 1:** FAME compositions in biodiesel derived from well-established feedstock.

FAME	BSFL (This Work at 0.5 mL) (%)	Soybean (%) [33]	Rapeseed (%) [33]	Oil Palm (%) [33]
C10:0	1.2	0.0	0.6	0.5
C12:0	47.5	0.1	0.1	0.3
C14:0	14.4	0.1	0.0	1.1
C16:0	14.6	11.6	4.2	42.5
C16:1	3.1	0.2	0.1	0.2
C18:0	2.1	3.9	1.6	4.2
C18:1	11.1	23.7	59.5	41.3
C18:2	4.0	53.8	21.5	9.5
C22:1	2.0	0.1	0.5	0.0
SFA	79.8	16.5	7.4	49.0
MUFA	16.2	24.7	62.8	41.6
PUFA (<4 π bonds)	4.0	59.7	30.0	9.8
Total	100.0	100.9	100.2	100.4

SFA: Saturated FAME; MUFA: Mono-unsaturated FAME; PUFA: Polyunsaturated FAME.

**Table 2:** Degradation rates of different organic substrates after black soldier fly larvae treatments.

Substrate	Degradation Rate (%)	Reference
Palm kernel expeller	38.8–62.4	This work
Coconut endosperm waste	52.0–75.0	[23,26]
Soybean curd residue	64.0–72.4	[26,34]
Cow manure	25.8	[34]
Corn stover	39.9	[35]
Fermented maize straw	48.4	[3]
Wheat bran	55.0	[3]
Fruits and vegetable waste	46.7–49.5	[2,36]
Food waste	50.3–55.3	[2,36]

## Data Availability

Not applicable.
